# Planctomycetes do possess a peptidoglycan cell wall

**DOI:** 10.1038/ncomms8116

**Published:** 2015-05-12

**Authors:** Olga Jeske, Margarete Schüler, Peter Schumann, Alexander Schneider, Christian Boedeker, Mareike Jogler, Daniel Bollschweiler, Manfred Rohde, Christoph Mayer, Harald Engelhardt, Stefan Spring, Christian Jogler

**Affiliations:** 1 Independent Junior Research Group Microbial Cell Biology and Genetics, Leibniz Institute-DSMZ, Inhoffenstraße 7b, Braunschweig 38124, Germany; 2 Department of Molecular Structural Biology, Max-Planck-Institute for Biochemistry, Am Klopferspitz 18, Martinsried 82152, Germany; 3 Department of Microbiology, Leibniz Institute-DSMZ, Inhoffenstraße 7b, Braunschweig 38124, Germany; 4 Department of Microbiology and Biotechnology, University of Tübingen, Auf der Morgenstelle 28, Tübingen 72076, Germany; 5 Research Group Molecular Mechanisms of Streptococci, Helmholtz Center for Infection Research GmbH, Inhoffenstraße 7, Braunschweig 38124, Germany

## Abstract

Most bacteria contain a peptidoglycan (PG) cell wall, which is critical for maintenance of shape and important for cell division. In contrast, Planctomycetes have been proposed to produce a proteinaceous cell wall devoid of PG. The apparent absence of PG has been used as an argument for the putative planctomycetal ancestry of all bacterial lineages. Here we show, employing multiple bioinformatic methods, that planctomycetal genomes encode proteins required for PG synthesis. Furthermore, we biochemically demonstrate the presence of the sugar and the peptide components of PG in Planctomycetes. In addition, light and electron microscopic experiments reveal planctomycetal PG sacculi that are susceptible to lysozyme treatment. Finally, cryo-electron tomography demonstrates that Planctomycetes possess a typical PG cell wall and that their cellular architecture is thus more similar to that of other Gram-negative bacteria. Our findings shed new light on the cellular architecture and cell division of the maverick Planctomycetes.

## Abstract (web summary)

Planctomycetes appear to differ from all other bacteria in their cellular organization and their apparent lack of a peptidoglycan (PG) cell wall. Here Jeske *et al*. show that Planctomycetes do possess a typical PG cell wall and that their cellular architecture resembles that of Gram-negative bacteria.

## Graphical abstract

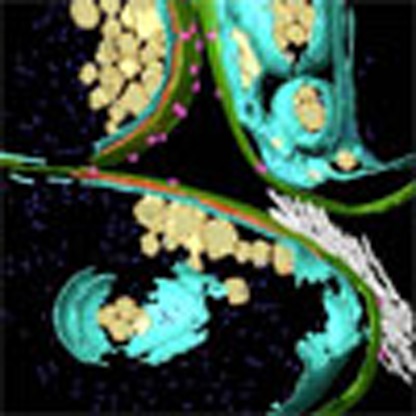

Dissolved solutes within free-living microorganisms cause significant osmotic pressure that challenges cell integrity. While most archaea rely on S-layers as protective exoskeletons^1^, bacteria possess peptidoglycan (PG) for maintaining their cell shape. PG is a rigid polymeric mesh of linear glycans^2^ cross-linked via short peptides^3^, whose remodelling—together with FtsZ and other proteins—is essential for septal formation during cell division^4^. Thus, most bacteria possess both, a PG cell wall and FtsZ, while the PG synthesis proteins and cell division proteins are encoded in the division and cell wall (*dcw*) operon. While members of the class Mollicutes lack PG, several species encode FtsZ^5^. However, Mollicutes are osmotically fragile, exhibit plasticity and pleomorphism^6^, and depend on an eukaryotic host to provide an osmotically stable environment for living. Thus, they are not considered as free-living. In contrast, cells of the free-living, ubiquitous and environmentally important phylum Planctomycetes were thought to lack PG and FtsZ as well^7^,^8^,^9^, and to possess a proteinaceous cell wall instead^10^,^11^. Indeed, the *dcw* clusters show signs of degradation in some Planctomycetes, where some genes are lost while other are present^8^,^9^. The absence of FtsZ may contribute to the unusual polar budding cell division of most Planctomycetes^7^. The inability to identify PG formed a cornerstone in the concept of a planctomycetal cell architecture that differs from all other bacteria. It was further proposed that their cytosol is divided by an additional intracytoplasmic membrane system into a paryphoplasm and a pirellulosome^7^, a concept that has been challenged recently employing bioinformatic and microscopic methods that pointed towards a more Gram-negative-like planctomycetal cell plan^12^,^13^,^14^. The question whether PG is present or absent in Planctomycetes is key for understanding (i) the compartmentalized planctomycetal cell architecture, (ii) the unusual FtsZ-independent cell division and (iii) the planctomycetal endocytosis-like uptake of proteins. Furthermore, this question is of significance in evolutionary terms as Planctomycetes were suggested as a ‘missing link' between prokaryotes and eukaryotes^15^ or as species ‘beyond the bacterium^7^. The postulation of a proteinaceous cell wall dates back three decades^10^ and has been passed on since then. Despite its conceptual importance, the cell wall composition of Planctomycetes has been rarely addressed experimentally^11^,^16^,^17^. However, recent reports on penicillin sensitivity of anammox Planctomycetes questioned their lack of a PG cell wall^18^. Furthermore, employing state-of-the-art methods, Chlamydiae were recently found to produce PG and a long lasting dispute about the ‘Chlamydiae anomaly' was settled^19^,^20^.

In this study, we revisit the nature of the planctomycetal cell wall. Employing modern bioinformatic approaches, we demonstrate that planctomycetal genomes harbour the genes required for PG synthesis. In addition, biochemical assays reveal the sugar and peptide components of PG in selected planctomycetal species. Our findings are further supported by light- and electron microscopic experiments that reveal planctomycetal cells and PG sacculi, both being susceptible to lysozyme treatment. Finally, cryo-electron tomography (CET) demonstrates that Planctomycetes possess a cell wall, comparable to that of other Gram-negative bacteria.

## Results

### Bioinformatic analysis

We re-analysed the planctomycetal phylogeny of the 16S rRNA gene sequences from selected species, including the novel, deep-branching, obligate anaerobic and halophilic strain L21-RPul-D3 (Supplementary Table 1), employing Maximum Likelihood, Neighbour Joining and Maximum Parsimony algorithms. The resulting phylogenetic tree (Supplementary Fig. 1) guided the selection of suitable model organisms for bioinformatic, biochemical and microscopic analyses, while its long branches might correspond to a high evolutionary divergence of cultivated planctomycetal species (see Supplementary Fig. 1, and Supplementary Note 1 for details).

In a second step, we employed comparative genomics to analyse planctomycetal genomes with respect to genes required for PG synthesis. Taking the assumed large evolutionary divergence of Planctomycetes into account, we used methods apt to deal with evolutionarily more distant sequences, and analysed entire planctomycetal genomes employing dynamic queries and position-specific iterative BLAST searches^17^ (see Supplementary Note 2 for details). Thus, contrary to a previous study that reported the absence of genes required for PG synthesis in some planctomycetal genomes^17^, we found that all the analysed species harbour essential genes for PG synthesis (Supplementary Tables 1 and 2, Supplementary Data 1-4 and Supplementary Discussion).

Another main argument for the absence of a PG cell wall has been the planctomycetal resistance to beta-lactam antibiotics, which target PG biosynthesis^10^. In an earlier study^17^, an alternative cause for the observed resistance, the production of beta-lactamases, has been ruled out for Planctomycetes. However, the employed nitrocefin-based activity test for beta-lactamases is not reliable^21^ (Supplementary Fig. 3 and Supplementary Note 3). Thus, we addressed the question of beta-lactam inactivating enzymes in Planctomycetes again, this time from a genomic perspective. We first identified representative proteins belonging to the beta-lactamase molecular classes A–D (Supplementary Table 3). With these sequences as queries, while employing very strict thresholds (see Supplementary Note 3 and Supplementary Discussion for details), we found at least one homologue in all the selected planctomycetal genomes (Supplementary Tables 4 and 5).

Thus, Planctomycetes have the genomic potential to synthesize PG, and also to produce beta-lactamases that could confer resistance against beta-lactam antibiotics.

### Biochemical analysis of the planctomycetal cell wall

**Figure 1:**
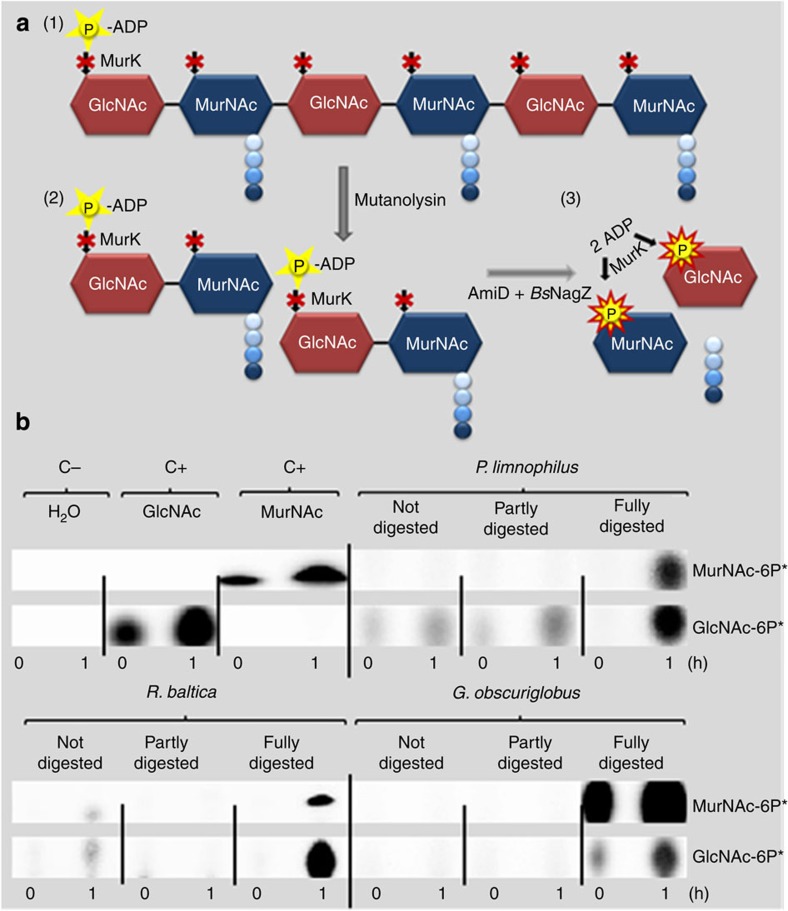
Radioactive labelling and detection of the PG sugars GlcNAc and MurNAc by TLC. (**a**) Principle of the radioactive kinase assay: polymeric PG is first partly digested by mutanolysin into disaccharides (stem peptides are still linked to MurNAc). The GlcNAc/MurNAc kinase MurK is not able to transfer phosphate (P) from [γ-^32^P]-ATP to polymers (1) and disaccharides (2) and thus, no signal is detected in TLC. Subsequently, the disaccharides are further digested into monomers and stem peptides (3). MurK is able to transfer the [γ-^32^P] from [γ-^32^P]-ATP to the sugar monomers GlcNAc and MurNAc and thus, a radioactive signal can be detected by TLC. (**b**) Radioactive detection of GlcNAc and MurNAc in either undigested, partly digested (treatment with mutanolysin) or fully digested (treatment with mutanolysin, AmiD and *Bs*NagZ) preparations of *P. limnophilus, R. baltica* and *G. obscuriglobus* cells on incubation with [γ-^32^P]-ATP and MurNAc/GlcNAc kinase MurK and subsequent TLC separation. Shown are time 0 and 1 h of incubation with [γ-^32^P]-ATP. C−: water negative control; C+: GlcNAc and MurNAc standards as positive controls.

In all bacteria the glycan part of PG consists of alternating residues of the *β*-1,4-linked amino sugars *N*-acetylglucosamine (GlcNAc) and *N*-acetylmuramic acid (MurNAc) (Fig. 1a; for a review see refs 2, 22). To detect these sugar monomers, we employed a radioactive kinase assay to analyse cells of three representative planctomycetal species (*Planctomyces limnophilus*, *Gemmata obscuriglobus* and *Rhodopirellula baltica*). This assay is based on the specific MurK-mediated transfer of the radioactively labelled γ-phosphoryl group from [γ-^32^P]-ATP exclusively to the 6-position of GlcNAc and MurNAc monomers, yielding GlcNAc-6-phosphate and MurNAc-6-phosphate, respectively^23^. Subsequent separation by thin-layer chromatography and detection via autoradiography revealed no signals for either of the sugar monomers when cells were not treated. The same result was obtained if PG was only partially digested with mutanolysin, a PG hydrolase like lysozyme, that cleaves the MurNAc-β-(1,4)-GlcNAc linkage of PG^24^ (Fig. 1b). In contrast, after full digestion with mutanolysin, the amidases AmiD and the *N*-acetylglucosaminidase *Bs*NagZ, both sugar components were detected in all the three investigated Planctomycetes, demonstrating the presence of MurNAc and GlcNAc in polymeric glycan strands (Fig. 1b).

**Figure 2:**
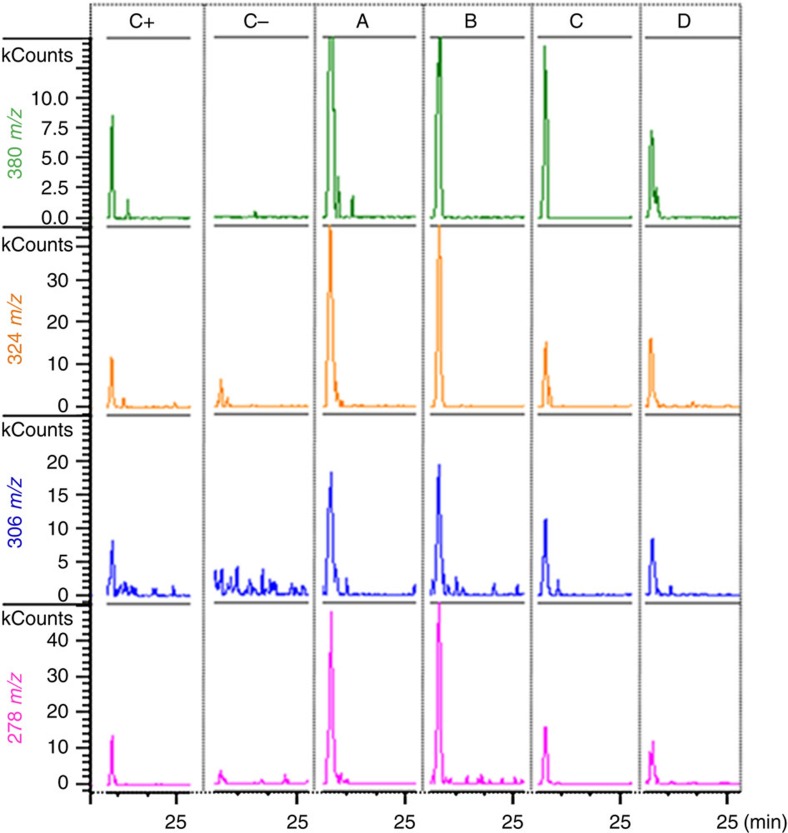
Mass spectrometric detection of the cell wall component DAP in planctomycetal cells. Ion chromatograms of the DAP derivative (*N*-heptafluorobutyryl DAP isobutylester) from whole-cell hydrolysates of: **C+**: *E. coli* K12 as positive control; **C−**: *Spirochaeta asiatica* DSM 8901 (ornithine-based PG) as negative control; **A**: *P. limnophilus* DSM 3776^T^; **B**: *G. obscuriglobus* DSM 5831^T^; **C**: *R. baltica* DSM 10527^T^; **D**: L21-RPul-D3. All the planctomycetal hydrolysates show peaks for specific masses (380, 324, 306 and 278 *m/z*) and retention time (23.7 min) of DAP fragments.

In most Gram-negative bacteria these glycan strands are cross-linked via stem peptide chains by the non-proteinogenic 2,6-diaminopimelic acid (DAP), which serves as trifunctional linker, typically at the third position in stem peptides^22^. The only well-documented exceptions are found among members of the *Spirochaetaceae* that use ornithine instead of DAP for cross-linking PG (for example, refs 25, 26). Consequently, the absence of DAP does not necessarily correspond to the absence of PG, while the presence of DAP is a strong indicator for cross-linked PG in bacteria. We analysed three planctomycetal model species together with strain L21-RPul-D3 for the presence of DAP by gas chromatography/mass spectrometry (GC/MS). All four investigated planctomycetal cell hydrolysates showed the specific fragment ion set for DAP at the respective retention time in the extracted ion chromatograms. The signals were significantly stronger than in *Spirochaeta asiatica* that served as a negative control (Fig. 2). The observed low levels of DAP (<2.5 kCounts) in our negative controls can be explained by the *de novo* synthesis of lysine in which DAP is synthesized as intermediate metabolite^27^.

In conclusion, our biochemical analyses provide an evidence for the existence of PG in Planctomycetes.

### Microscopic analyses of planctomycetal cell walls

**Figure 3:**
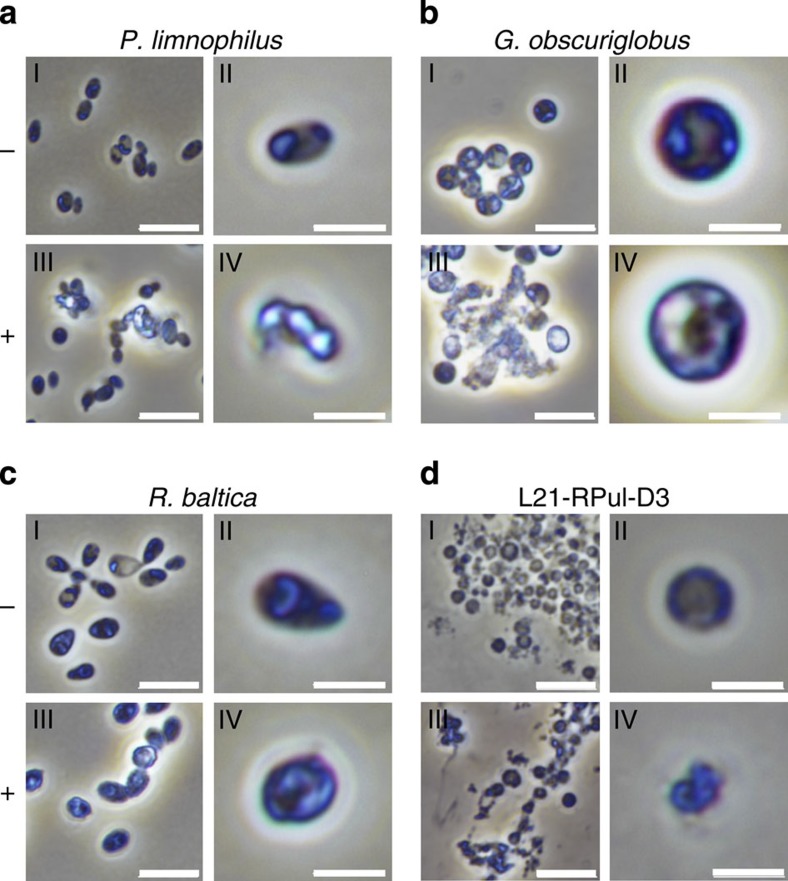
Effect of lysozyme on planctomycetal cells. Phase-contrast micrographs of (**a**) *P. limnophilus*, (**b**) *G. obscuriglobus*, (**c**) *R. baltica* and (**d**) L21-RPul-D3 cells after lysozyme treatment (III and IV). Untreated cells (I and II) serve as negative control. Scale bars, 5 μm (I, III) and 2 μm (II, IV).

Lysozyme treatment leads to cell disruption by hydrolysis of the *β*-1,4-linkages between MurNAc and GlcNAc^28^. Images of untreated Planctomycetes show subcellular details under phase-contrast illumination that potentially correspond to the planctomycetal cell compartmentalization (Fig. 3a-d, panels I and II). After lysozyme treatment, the cells lose these details along with their shape or lyse entirely (Fig. 3a-d, panels III and IV). Thus, lysozyme disrupts the planctomycetal cell structure.

**Figure 4:**
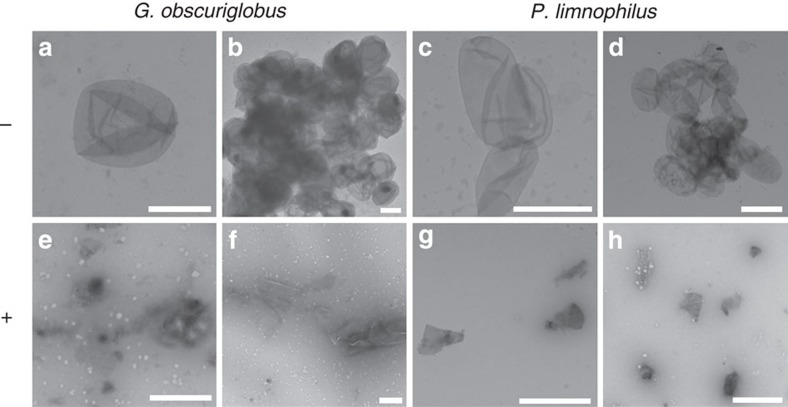
Effect of lysozyme on planctomycetal PG sacculi. TEM micrographs of planctomycetal cell sacculi prepared after 3 h of boiling in 10% SDS and purification through ultracentrifugation. Sacculi of *G. obscuriglobus* DSM 5831^T^ (**a**,**b**,**e**,**f**) and *P. limnophilus* DSM 3776^T^ (**c**,**d**,**g**,**h**) were untreated (**a**,**b**,**c**,**d**) or incubated with lysozyme (**e**,**f**,**g**,**h**) before negative staining with 0.1%–2% aqueous uranyl acetate. Scale bars, 1 μm.

Cell wall sacculi were prepared from *P. limnophilus* and *G. obscuriglobus* and purified from lipid and protein by treatment in a boiling SDS solution. Both species yielded intact sacculi of cell size (Fig. 4a–d) that resemble PG sacculi of other Gram-negative bacteria in shape and appearance^29^. Treatment of isolated sacculi with lysozyme led to disintegration and fragmentation of the sacculi structures (Fig. 4e–h). Moreover, we could verify the presence of DAP in *P. limnophilus* sacculi by employing our GC/MS detection method (Supplementary Fig. 2).

**Figure 5:**
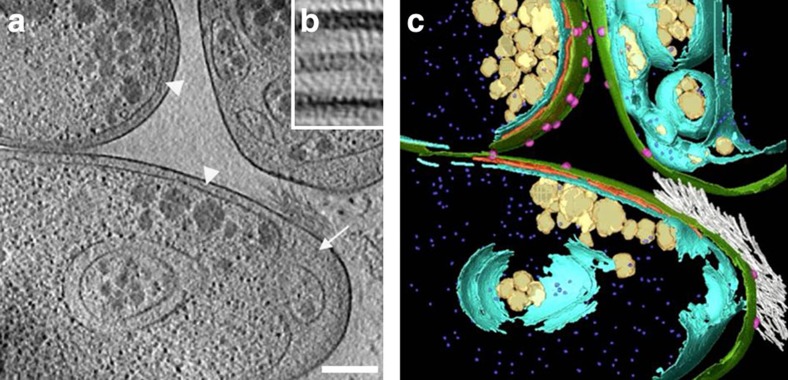
CET of *P. limnophilus* cells. (**a**) Tomographic slice from a vitrified, cryo-thinned *P. limnophilus* sample showing the pole regions of three adjacent cells. A cell wall layer between the outer and inner membrane is visible in regions where the two membranes run parallel (arrowheads). The layer remains close to the outermost membrane where the inner membrane is invaginated (arrow). The reconstruction is binned three times and filtered. Scale bar, 200 nm. (**b**) Average of 1,059 subframes extracted from tomographic *x*–*y* slices along the cell envelope from non-polar regions where the inner membrane (bottom) runs approximately in parallel to the outer one (top). The inner membrane is only imperfectly aligned because of its varying distance to the outer membrane (also visible in **a**). The cell wall layer in between is clearly detectable (centre-to-centre distance to the outer membrane ≈22 nm). Contrary to the inner and outer membrane layers, which show high electron density, typically originating from phosphate in lipid head groups, the PG layer produces less contrast. Size of image 45 nm × 65 nm. (**c**) Segmentation of the reconstructed volume. Green: external membrane, cyan: internal membrane, red: PG layer, magenta: crateriform structures, violet: ribosomes, yellow: storage granules and white: holdfast substance.

*P. limnophilus* cells vitrified and thinned by focused ion beam milling^30^, and investigated under near-native conditions by CET showed an electron-dense layer between the external and the internal membrane (Fig. 5 and Supplementary Movie 1). This layer could be seen in cells from different growth phases and in all the 25 tomograms inspected. The location and somewhat fuzzy appearance is typical for a thin PG network of Gram-negative cells^31^. The optical density of the PG layer is generally poor in the polar regions and in slices that are distant from the cell's central plane (this is an effect of the oblique orientation of the PG with respect to the *x*–*y* slice, and of the ‘missing wedge'^31^). However, the cell wall layer can be detected in tomographic slices from central cell regions and particularly clearly in averaged subframes of the cell envelope (Fig. 5b).

## Discussion

Planctomycetes belong together with Verrucomicrobia and Chlamydiae to the PVC superphylum^32^, an unusual group of bacteria that comprise several exceptional traits^33^. Besides Chlamydiae^19^, Planctomycetes and certain Verrucomicrobia (for example ref. 34) were thought to lack a PG cell wall. However, contrary to the longstanding belief, we found that Planctomycetes do possess a rigid PG cell wall.

All our results are in agreement with what is known about typical constituents and features of PG from Gram-negative bacteria. Aside from specific biochemical and enzymatic evidence, we were able to isolate intact PG sacculi by harsh treatment, and could show the existence of a thin (≤10 nm) cell wall layer between the outer and inner planctomycetal membranes, that is the basic architecture of cell envelopes from Gram-negative bacteria. We assume that the PG sacculi completely cover the cells but can currently not exclude that local or temporary discontinuities occur.

Our results put the principal hypothesis on the uniqueness of planctomycetal cellular structure to test and are in line with a number of other recent findings^12^,^13^,^14^. For instance, presence of an asymmetric outer membrane layer in Planctomycetes was suggested^14^ and the report on the 3D structure of *G. obscuriglobus* cells^13^ challenged the current view of its compartmentalization^7^. In this context, the existence of a PG layer, as shown in this study, is thus an important aspect as it strongly supports the ‘classical' Gram-negative organization of the planctomycetal cell envelope. Whether the massive invaginations of the inner membrane observed in our tomograms of *P. limnophilus* may result in closed compartments other than a single cytoplasmic volume –as recently suggested for *G. obscuriglobus*^13^—remains to be investigated in detail.

Furthermore, the existence of a PG layer in Planctomycetes has implications with respect to the endocytosis-like protein uptake in Planctomycetes^35^. Vesicles of 50 to 200 nm in size, which are thought to originate from the outer planctomycetal membrane by invagination^35^, could be blocked by a PG layer with a typical mesh size of 1.6–2.0 nm (ref. 29).

Future studies have to elucidate whether the planctomycetal PG comprises pores or if the functional concept of endocytosis in Planctomycetes must be reconsidered.

The results of this study mark Planctomycetes as second phylum after Chlamydiae^19^, in which simultaneous presence of PG and absence of the otherwise universal and essential bacterial cell division protein FtsZ was observed. A study on L-form *Bacillus* strains demonstrated that FtsZ became non-essential in cells growing without a wall^36^. This study further suggested that Planctomycetes might use the same—potentially ancient—way of division^36^. However, our work and the lack of FtsZ among Planctomycetes^8^,^9^ demonstrate that bacterial cell division without FtsZ does not correlate with the absence of a PG cell wall. Thus, a fundamentally different molecular mechanism of planctomycetal cell division through budding might exist. The recent finding of PG in Chlamydiae that lack FtsZ as well^19^, further supports this interpretation and bacterial cell division might be more diverse than currently appreciated.

Given that Planctomycetes (this study), anammox Planctomycetes (van Teseling *et al*.^52^) and Chlamydiae^19^,^20^ contain PG cell walls, the question remains whether certain Verrucomicrobia are really exceptional in this aspect^34^. The methods used and developed in this study will be instrumental to address this question in the future.

In conclusion our findings suggest that, despite several unique features, Planctomycetes are more Gram-negative-like than previously proposed^7^.

## Methods

### Phylogeny

Gene sequences (16S rRNA; Supplementary Table 1) were aligned (SINA Alignment Service^37^) and manually corrected. Maximum Likelihood (RAxML module; rate distribution model GTRGAMMA; rapid bootstrap analysis algorithm), NJ (ARB NJ tool; Felsenstein correction) and Maximum Parsimony (Phylip DNAPARS module) trees were calculated including the *E. coli* 16S rRNA gene positions 63–1,406, employing the ARB software package^38^. Bootstrap values were computed with 1,000-fold resampling.

### Beta-lactamase assay

Beta-lactamase production was triggered for 2 h (100 μg ml^−1^ ampicillin). Cells were then lysed on ice by sonication (2 × 2 min, 70%; Bandelin, Sonopuls) and pelleted using a centrifuge. Then 50 μl nitrocefin (0.5 mg ml^−1^; Calbiochem) were added to 500 μl of the supernatant. After 30 min incubation in the dark a red colour indicated beta-lactamase activity.

### Bioinformatic analysis of planctomycetal genomes

Proteins required for PG synthesis from *E. coli* were used as blastp^39^ and PSI-BLAST (five iterations^40^) query to identify homologues in *P. limnophilus* and *P. brasiliensis.* The obtained planctomycetal proteins were then compared against all the other sequenced Planctomycetes (Supplementary Table 1 and 2 and Supplementary Data 2-4). Homologues required a threshold (identity >30% and an *e* value <1*e*-6), conserved domain architecture^41^ and a reciprocal blastp match against *E. coli*.

Beta-lactamase enzymes (selected based on ref. 42, see Supplementary Table 3) were compared against the NCBI database using blastp^40^ with default settings and ‘Planctomycetes' as taxon filter. Homologues were identified using an initial threshold (identity⩾20%; coverage⩾40%; *e* value≤*e*-4) and a reciprocal blast analysis (identity⩾30%; coverage⩾45%; *e* value≤*e*-6).

### Cultivation conditions

Unless noted otherwise, *Rhodopirellula baltica* SH1 DSM 10527 was cultured at 28 °C in M2 medium composed of 1 g l^−1^ peptone, 1 g l^−1^ glucose, 250 ml double-concentrated artificial sea water (46.94 g l^−1^ NaCl, 7.84 g l^−1^ Na_2_SO_4_, 21.28 g l^−1^ MgCl_2_ × 6H_2_O, 2.86 g l^−1^ CaCl_2_ × 2H_2_O, 0.384 g l^−1^ NaHCO_3_, 1.384 g l^−1^ KCl, 0.192 g l^−1^ KBr, 0.052 g l^−1^ H_3_BO_3_, 0.08 g l^−1^ SrCl_2_ × 6H_2_O, 0.006 g l^−1^ NaF), 10 ml vitamin solution (double concentrated) and 20 ml l^−1^ mineral salt solution buffered with 5 mM Tris/HCl at pH 7.5. *Planctomyces limnophilus* DSM 3776 and *Gemmata obscuriglobus* DSM 5831 were cultivated at 28 °C in M3 medium composed of 1 g l^−1^ peptone, 1 g l^−1^ yeast extract, 1 g l^−1^ glucose, 5 ml vitamin solution (double concentrated) and 20 ml l^−1^ mineral salt solution buffered with 10 mM HEPES at pH 7.5. Strain L21-RPul-D3 was obtained from the anoxic zone of a hypersaline microbial mat on the Kiritmati atoll (Central Pacific). Cells of this strain were cultured anaerobically at 35 °C for 6 days in DSMZ medium 1527 in which D-glucose was replaced with 0.5 g l^−1^ dextran as a carbon source. Mineral salt solution and vitamin solution were prepared according to DSMZ medium 621. The metal salts for preparing mineral salt solution consisted of 250 mg l^−1^ Na-EDTA, 1095, mg l^−1^ ZnSO_4_ × 7H_2_O, 500 mg l^−1^ FeSO_4_ × 7H_2_O, 154 mg l^−1^ MnSO_4_ × H_2_O, 39.5 CuSO_4_ × 7H_2_O mg l^−1^, 20.3 mg l^−1^ CoCl_2_ × 6H_2_O, 17.7 mg l^−1^ Na_2_B_4_O_7_ × 10H_2_O of which 50 ml were added per litre of mineral salt solution. *Escherichia coli* cells DSM 498 were grown over night at 37 °C in lysogeny broth (LB). *Spirochaeta asiatica* DSM 8901 was cultivated for 2 days in DSMZ medium 1263 at 35 °C. For the detection of diaminopimelic acid, *P. limnophilus* was cultivated in 10 μM NH_4_Cl, 10 μM KH_2_PO_4_, 100 μM KNO_3_, 200 μM MgSO_4_ × 7H_2_O, 100 μM CaCl_2_ × 2H_2_O, 250 μM CaCO_3_ and 300 μM NaHCO_3_, 20 ml l^−1^ mineral salts solution and *R. baltica* was cultured in 250 ml l^−1^ of 2 × artificial sea water (46.94 g l^−1^ NaCl, 7.84 g l^−1^ Na_2_SO_4_, 21.28 g l^−1^ MgCl_2_ × 6H_2_O, 2.86 g l^−1^ CaCl_2_ × 2H_2_O, 0.384 g l^−1^ NaHCO_3_, 1.384 g l^−1^ KCl, 0.192 g l^−1^ KBr, 0.052 g l^−1^ H_3_BO_3_, 0.08 g l^−1^ SrCl_2_ × 6H_2_O and 0.006 g l^−1^ NaF) and 5 ml l^−1^ 1M Tris/HCl pH 7.5). Each culture broth was supplemented with 0.25 g l^−1^ glucose. *G. obscuriglobus* cells were grown like *P. limnophilus*, but supplemented with 0.25 g l^−1^ pullulan instead of glucose. Cultures were incubated at 28 °C for at least 3 days. *S. asiatica* was grown in DSMZ medium 1263 at 35 °C for 2 days. For MurNAc and GlcNAc detection *P. limnophilus* medium was supplemented with 0.25 g l^−1^ dextran instead of glucose at 28 °C for 3 days. *M. vannielii* was cultivated in DSMZ medium 119 at 35 °C for 2 days.

### Detection of GlcNAc and MurNAc

Cell pellets from 50 ml culture were treated with substrate-specific PG-hydrolysing enzymes (*Streptomyces globisporus* mutanolysin from Sigma, *E. coli* MurNAc-L-alanyl amidase D, AmiD, isolated according to ref. 43, *Bacillus subtilis N*-acetylglucosaminidase, *Bs*NagZ, isolated according to ref. 44; 10 μg enzyme were used each, per 40 μl sample) and then incubated with 80 ng of the MurNAc/GlcNAc specific kinase of *Clostridium acetobutylicum*, MurK, isolated according to ref. 41 and [**γ**-^32^P]-ATP (3,000 Bq). Samples were spotted immediately and after 1 h of incubation on a thin-layer chromatography (TLC) plate. The TLC was developed in a solvent mixture of *n*-butanol, methanol, 25% aqueous NH_3_ and water (5:4:2:1) and detected after incubation for 12 h using a photosensitive screen (Kodak) on a Typhoon FLA7000 biomolecular phosphoimager (GE Healthcare).

### Detection of diaminopimelic acid

Cell pellets were obtained from 6–20 ml cultures, lyophilized, hydrolysed (200 μl 4 N HCl, 100 °C, 16 h) and dried in a vacuum desiccator. Amino-acids derivatized to *N*-heptafluorobutyryl isobutylesters^45^ were resolved in ethyl acetate and analysed by GC/MS (Singlequad 320, Varian; electron impact ionization, scan range 60 to 800 *m/z*). The derivatized diaminopimelic acid was detected in Extracted Ion Chromatograms using the characteristic fragment ion set 380, 324, 306 and 278 *m/z* at a retention time of 23.7 min.

### Lysozyme assay

To increase osmotic stress, *P. limnophilus* and *G. obscuriglobus* cells were washed with ddH_2_O, while *R. baltica* and L21-RPul-D3 were washed in tap water and artificial sea water, respectively. Cells were incubated with EDTA (20 mM) and lysozyme (10 mg ml^−1^) for up to 48 h at 37 °C and shaking (300 r.p.m.), while lysozyme was omitted in the negative controls. Cells were immobilized (1% agarose-pad) in MatTek 35-mm glass-bottom dishes and imaged under phase-contrast illumination using a Nikon Ti microscope at × 100 magnification and the Nikon DS-Ri2 camera employing the Nikon NIS-Elements software.

### TEM analysis of planctomycetal PG sacculi

*G. obscuriglobus* DSM 5831^T^ and *P. limnophilus* DSM 3776^T^ were cultivated in 1 l of respective growth media. Cells were harvested (10 min, 4,000 *g*, 4 °C; JA-14 rotor, Beckman Coulter) and cell pellets were resuspended in 10 ml 10% SDS (w/v) and boiled for 3 h. Sacculi were harvested (30 min, 139,699 *g*, 4 °C; SW 60 Ti rotor, Beckman Coulter), washed four times in 3 ml water and resuspended in 1 ml water supplemented with 0.02% (w/v) sodium azide. Half of the prepared sacculi were incubated with lysozyme (100 mg ml^−1^) for 2 h at 37 °C under slight agitation (300 r.p.m.), while lysozyme was omitted in the negative controls. TEM micrographs of murein sacculi were taken after negatively staining with 0.1–2% aqueous uranyl acetate, employing a Zeiss transmission electron microscope EM 910 at an acceleration voltage of 80 kV at calibrated magnifications as previously described^46^.

### CET of *P. limnophilus* cells

A late-exponential-phase culture of *P. limnophilus* was gently filtered (10 μm membrane filter, Whatman Nuclepore) to remove aggregated cells. Aliquots (3 μl) of the filtered cell suspension were mixed with the same volume of BSA-stabilized 15 nm colloidal gold solution (Aurion) and placed on holey-carbon coated 200 mesh copper grids (R2/1, Quantifoil, Jena, Germany) immediately before thin-film vitrification by plunge-freezing in liquid propane (63%)/ethane (37%). Grids with frozen-hydrated samples were mounted in autogrids^47^ and ∼200 nm thin lamellae of vitrified material were milled with 30 keV gallium ions after the application of a protective platinum layer in a dual-beam (FIB/SEM) instrument (Quanta 3D FEG, FEI, Hillsboro, OR, USA) equipped with a Quorum cryo-stage maintained at −185 °C (PP2000T, Quorum, East Sussex, UK). Milling was carried out at a nominal incident ion beam angle of 22° (15° effectively) using gallium beam currents of 300, 100 and 30 pA in sequential milling steps. Afterwards tomographic tilt series were recorded under low-dose conditions (total dose typically 150 e Å^−2^) on a Titan Krios II (FEI, Hillsboro, OR, USA) equipped with a post-column energy filter and a K2 summit direct electron detector (Gatan, Pleasanton, CA, USA). In the exemplary tomogram (Fig. 5) an angular range from −68° to 54° was covered in 2° increments, and the tilt series was recorded at a nominal defocus of −5 μm and a primary magnification of × 33,000 (that is, a pixel size of 0.42 nm on the object level). Dose fractionation mode was employed and subframes of each projection were sampled, which were then aligned using custom-made scripts. Estimation and correction of the contrast transfer function was performed for the frame-corrected set of projections according to ref. 48, followed by 3D reconstruction in IMOD v4.7.8, using patch tracking for fine alignment since not enough gold markers were present after sample thinning. Cell wall cross-section averaging for density profile calculation was done on a 3D data set (binned once; 0.85 nm per pixel) as described in ref. 49. Subframes (90 × 90 pixels) of the cell envelope were extracted from 17 *x–y* slices central to the *z*-direction of a tomogram, aligned and averaged. MatLab8 (MathWorks) incorporating the TOM toolbox^50^ was used for all image processing.

Prior to segmentation, the 3D volume was binned three times and filtered by nonlinear anisotropic diffusion in IMOD (*K* value 0.03, 20 iterations). Segmentation in general was done in Amira v5.6.0 with specific automatic membrane segmentation according to ref. 51.

## Author contributions

O.J. and C.J. designed the project. S.S. isolated strain L21-RPul-D3. O.J. and S.S. cultivated all the required bacteria with the help from M.J.. M.J. performed the phylogenetic analysis and the bioinformatic screen for beta-lactamases. O.J. identified PG-related genes. A.S. and C.M. detected the cell wall sugars with a radioactive kinase assay. P.S. measured DAP employing GC/MS with help from O.J. and S.S. C.B. treated the cells with lysozyme and performed light-microscopic observations. O.J. prepared sacculi that were imaged with TEM by M.R. M.S. performed CET experiments and M.S., D.B. and H.E. analysed the data. O.J. and C.J. wrote the manuscript with the help from M.S. and H.E. and the input from all the other authors.

## Additional information

**How to cite this article**: Jeske, O. *et al*. Planctomycetes do possess a peptidoglycan cell wall. *Nat. Commun.* 6:7116 doi: 10.1038/ncomms8116 (2015).

## Supplementary Material

Supplementary Figures, Supplementary Tables, Supplementary Notes, Supplementary Discussion and Supplementary References Supplementary Figures 1-3, Supplementary Tables 1-5, Supplementary Notes 1-3, Supplementary Discussion and Supplementary References

Supplementary Movie 1 Tomographic reconstruction from a vitrified, cryo-thinned *P. limnophilus* sample showing the pole regions of three adjacent cells. A cell wall layer between the outer and inner membrane is visible in regions where the two membranes run parallel. The layer remains close to the outermost membrane where the inner membrane is invaginated. The reconstruction is binned three times and filtered. Green: external membrane, cyan: internal membrane, red: peptidoglycan layer, magenta: crateriform structures, violet: ribosomes, yellow: storage granules, white: holdfast substance.

Supplementary Data 1 Blastp-based analysis for the identification of peptidoglycan biosynthesis proteins in Planctomycetes employing *E. coli* proteins as query.

Supplementary Data 2 Blastp-based analysis for the identification of peptidoglycan biosynthesis proteins in Planctomycetes employing *P. limnophilus* proteins as query.

Supplementary Data 3 PSI-BLAST-based analysis for the identification of peptidoglycan biosynthesis proteins in Planctomycetes.

Supplementary Data 4 Reciprocal blastp based analysis of peptidoglycan biosynthesis proteins in Planctomycetes.
