# Supplementary material for: Planctomycetes do possess a peptidoglycan cell wall
Source: Nat Commun. 2015 May 12;6:7116. doi: 10.1038/ncomms8116 (PMC4432640; doi:10.1038/ncomms8116)
Supplement: Supplementary Figures, Supplementary Tables, Supplementary Notes, Supplementary Discussion and Supplementary References — Supplementary Figures 1-3, Supplementary Tables 1-5, Supplementary Notes 1-3, Supplementary Discussion and Supplementary References [file ncomms8116-s1.pdf]

Supplementary Information

1. Supplementary Figures

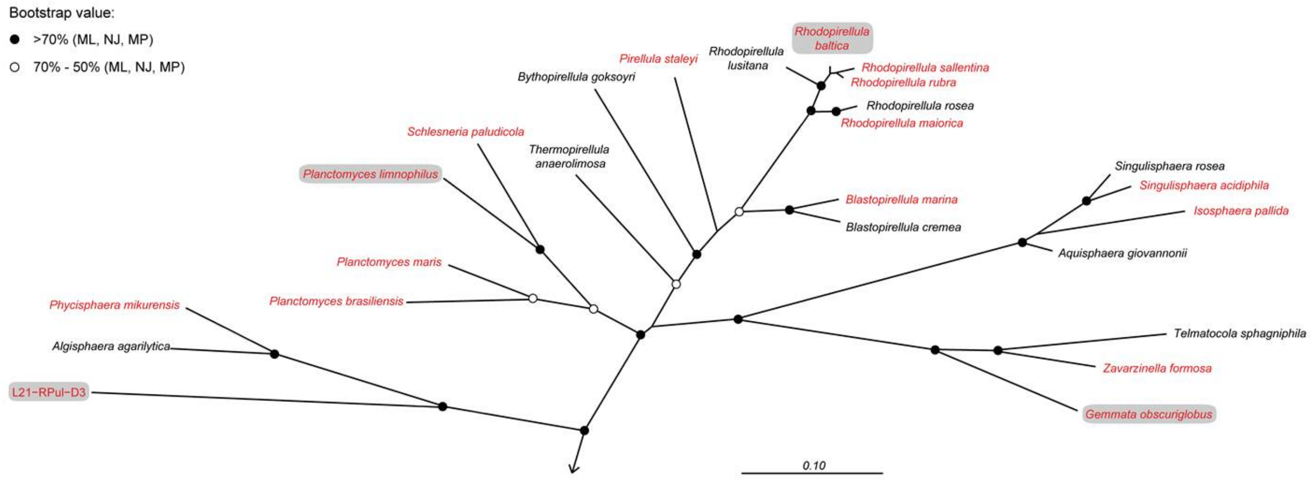

Supplementary Figure 1: Phylogenetic analysis of selected Planctomycetes

Schematic Maximum Likelihood phylogenetic tree of selected Planctomycetes based on the 16S rRNA gene sequence (for detailed information see Supplementary Table 1). Species with available genomes, used for bioinformatic analysis, are indicated in red. Species used for detailed experimental analysis in this study are highlighted in grey. Bootstrap values are based on 1000 resamplings with the Maximum Likelihood (ML), Neighbour Joining (NJ) and Maximum Parsimony (MP) algorithm. Selected anammox planctomycetes were used as out-group and are represented by an arrow ("*Candidatus* Brocadia anammoxidans", AF375994; "Candidatus Scalindua brodae", AY257181; "*Candidatus* Kuenenia stuttgartiensis", CT573071; "*Candidatus* Jettenia asiatica", DQ301513; "*Candidatus* Brocadia fulgida", DQ459989; "*Candidatus* Scalindua wagneri", EU478692; "*Candidatus* Anammoxoglobus propionicus", EU478694; Planctomycete KSU-1, AB057453).

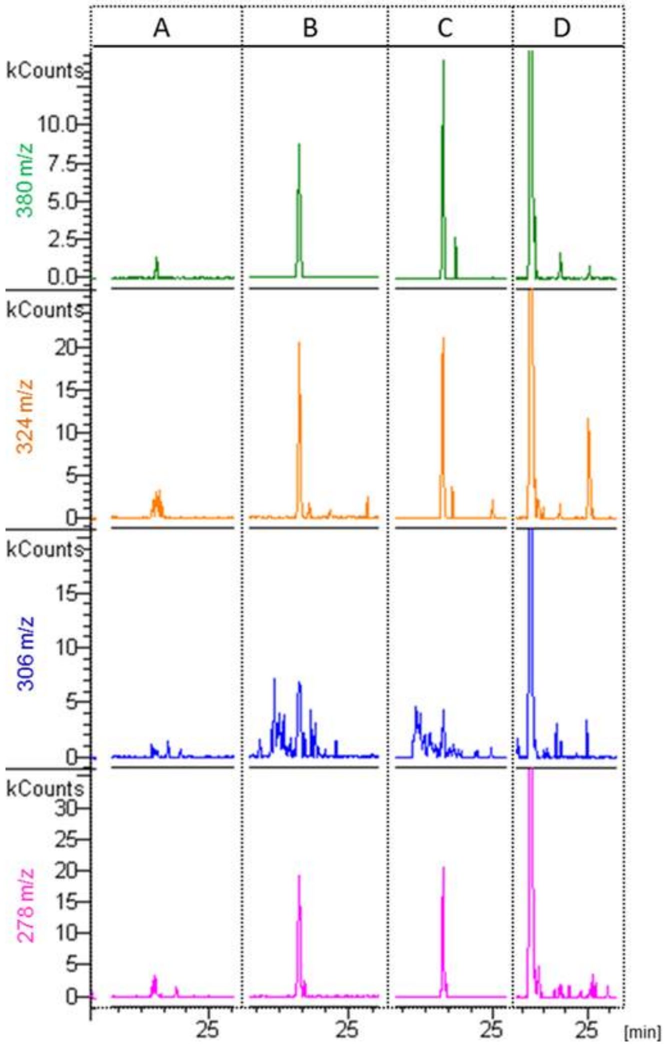

Supplementary Figure 2: Detection of 2,6-diaminopimelic acid (DAP) in Gram-positive and Gram- negative bacteria and *P. limnophilus* PG sacculi.

Extracted Ion Chromatograms (EIC) of the DAP derivative (*N*-heptafluorobutyryl 2,6-diaminopimelic acid isobutylester) from murein sacculi hydrolysates. **A:** Standard cell wall mix solution (12 pmol/µl DAP) as positive control; **B:** *Catenibacterium mitsuokai* DSM 15897 as a Gram-positive control; **C:** *E. coli* DSM 498 as a Gram-negative control; **D:** *P. limnophilus* PG sacculi. The *P. limnophilus* PG sacculi hydrolysate shows the peaks for specific masses (380 m/z, 324 m/z, 306 m/z, 278 m/z) of DAP fragment ions and retention time (23.7 min) of the DAP derivative.

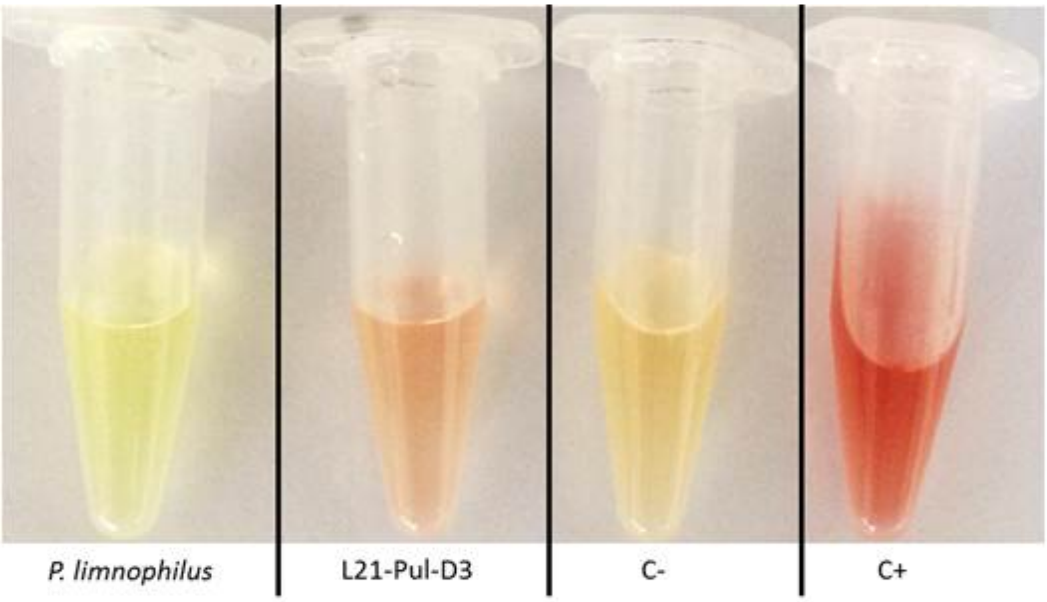

Supplementary Figure 3: Nitrocefin hydrolysis beta-lactamase activity assay. Supernatant of lysed cells was treated with 50 µl nitrocefin (0.5 mg/mL). Hydrolysis of nitrocefin produces a wavelength shift from intact yellow nitrocefin (~380 nm) to degraded red nitrocefin (~500 nm). C+ positive control: *E. coli* with ampicillin resistance cassette; C- negative control: the archaeon *Methanococcus vannielii* that lacks any β-lactamase.

2. Supplementary Tables

Species	Strain	Genome accession number	16S rRNA gene accession number
Algisphaera agarilytica	06SJR6-2	-	AB845176
Aquisphaera giovannonii	OJF2^T	-	NR_122081
Bythopirellula goksoyri	Pr1d	-	NR_118636
Blastopirellula cremea	LHWP2^T	-	NR_118153
Blastopirellula marina	SH 106^T	NZ_AANZ00000000	NR_029226
Gemmata obscuriglobus	UQM 2246^T	NZ_ABGO00000000	NR_114712
Isosphaera pallida	IS1B^T	NC_014962.1	NR_074534
L21−RPul−D3	L21−RPul−D3	unpublished	KC665947
Phycisphaera mikurensis	FYK2301M01^T	NC_017080.1	NR_074491
Pirellula staleyi	ATCC 27377^T	NC_013720.1	NR_074521
Planctomyces brasiliensis	DSM 5305^T	NC_015174.1	NR_074297
Planctomyces limnophilus	Mü 290^T	NC_014148.1	NR_074670
Planctomyces maris	534-30^T	NZ_ABCE00000000	NR_025327
Rhodopirellula baltica	SH 1^T	NC_005027.1	NR_043384
Rhodopirellula lusitana	UC17	-	EF589351
Rhodopirellula maiorica	SM1	ANOG00000000	FJ624363
Rhodopirellula rosea	LHWP3	-	JF748734
Rhodopirellula rubra	SWK7	ANOQ00000000	FJ624377
Rhodopirellula sallentina	SM41	ANOH00000000	FJ624360
Schlesneria paludicola	MPL7^T	NZ_AHZR00000000	NR_042466
Singulisphaera acidiphila	MOB10^T	NC_019892.1	NR_102439
Singulisphaera rosea	S26^T	-	NR_116969
Thermopirellula anaerolimosa	VM20-7	-	AB558583
Telmatocola sphagniphila	SP2^T	-	NR_118328
Zavarzinella formosa	A10^T	AIAB00000000	NR_042465

Supplementary Table 1: Planctomycetal reference sequences used in this study. Strains with > 99.5% 16S rRNA gene similarity to *Rhodopirellula baltica* SH1 were excluded from the analysis (*Rhodopirellula baltica* SH28, *Rhodopirellula baltica* SWK14, *Rhodopirellula baltica* WH47, *Rhodopirellula europaea* 6C, *Rhodopirellula europaea* SH398). ^{T} = type strain according to the List of Prokaryotic Names with Standing in Nomenclature (July 2014).

Protein/ Organism	MurA	MurB	MurC	MurD	MurE	MurF	MraY	MurG	MurJ/ MviN	pbp
Blastopirellula marina	+	+	(+)	(+)	+	(+)	+	+	+	+
Gemmata obscuriglobus	+	+	(+)	(+)	(+)	(+)	+	+	+	+
Isosphaera pallida	+	+	(+)	(+)	(+)	(+)	+	+	(+)	+
L21-Pul-D3	+	+	+	+	+	+	+	+	+	+
Phycisphaeara mikurensis	+	+	+	+	+	+	+	+	+	+
Pirellula staleyi	+	+	(+)	(+)	+	(+)	+	+	+	+
Planctomyces brasiliensis	+	+	+	+	+	+	+	+	+	+
Planctomyces limnophilus	+	+	+	+	+	+	+	+	+	+
Planctomyces maris	+	+	+	+	+	+	+	+	+	+
Rhodopirellula baltica SH1	+	+	(+)	(+)	+	(+)	+	+	-	-
Rhodopirellula maiorica	+	+	(+)	(+)	+	(+)	+	+	+	+
Rhodopirellula sallentina	+	-	(+)	(+)	+	(+)	+	+	(+)	-
Rhodopirellula rubra	+	+	(+)	(+)	+	+	+	+	+	-
Schlesneria paludicola	+	+	+	+	+	+	+	+	+	+
Singulisphaera acidiphila	+	+	+	(+)	+	(+)	+	+	+	+
Zavarzinella formosa	+	+	(+)	(+)	(+)	(+)	-	+	+	+

Supplementary Table 2: Presence or absence of predicted peptidoglycan biosynthesis proteins in planctomycetal genomes

+ : BLAST identity >30%, e-value < 1e-6, positive domain identification. In case of lower sequence identity positive domain identification was rated more important.

(+): BLAST identity >20% but <30%, e-value < 1e-4 but > 1e-6 positive domain identification.

- : No homolog detected above thresholds.

See supplementary Data 2-4 for details.

Class[1]	Species	Accession number
A	*Mycobacterium fortuitum*	Q59517
B1a	*Bacillus cereus* 569	P04190
B1b	*Klebsiella pneumoniae* 05-	C7C422
B2	*Aeromonas hydrophila*	P26918
B3	*Fluoribacter gormanii*	Q9K578
C	*Escherichia coli* K-12	P00811
D	*Acinetobacter baumannii*	Q9F0V3

Supplementary Table 3: List of representative beta-lactam proteins belonging to the molecular classes A-D and subclasses B1-B3 used for identification of putative beta-lactamases in Planctomycetes.

Species	(Sub-) Classes						
	A	B1a	B1b	B2	B3	C	D
Blastopirellula marina	-	-	-	1	-	5	-
Gemmata obscuriglobus	1	-	-	1	-	3	-
Isosphaera pallida	-	-	-	-	-	3	-
L21−RPul−D3	-	-	-	-	1	-	-
Phycisphaera mikurensis	-	-	-	-	-	1	-
Pirellula staleyi	-	-	-	-	-	4	-
Planctomyces brasiliensis	-	-	-	-	1	4	-
Planctomyces limnophilus	-	-	-	-	1	3	-
Planctomyces maris	-	-	-	1	1	5	-
Rhodopirellula baltica	-	-	-	2	-	4	-
Rhodopirellula maiorica	-	-	-	2	-	7	-
Rhodopirellula rubra	-	-	-	1	-	3	-
Rhodopirellula sallentina	-	-	-	2	-	-	-
Schlesneria paludicola	-	-	-	1	1	2	-
Singulisphaera acidiphila	1	-	-	-	2	16	-
Zavarzinella formosa	1	-	-	-	1	6	-

Supplementary Table 4: Number of beta-lactamase related proteins found within planctomycetal genomes. The analysis is based on blastp comparison with representative proteins belonging to the molecular classes A-D and subclasses B1-B3 (for details see Supplementary Table 5).

Species	Accession number	Class
Blastopirellula marina	WP_002651568.1	C
	WP_002655319.1	C
	WP_002650735.1	C
	WP_002654799.1	C
	WP_002651310.1	C
	WP_002654004.1	B2
Gemmata obscuriglobus	WP_029601377.1	A
	WP_010043637.1	C
	WP_029600663.1	C
	WP_010035646.1	C
	WP_010049328.1	B2
Isosphaera pallida	WP_013564050.1	C
	WP_013566120.1	C
	WP_013566464.1	C
L21-RPul-D3	6666666.51128.peg.1253[2]	B3
Phycisphaera mikurensis	WP_014437849.1	C
Pirellula staleyi	WP_012910543.1	C
	WP_012909960.1	C
	WP_012912429.1	C
	WP_012910255.1	C
Planctomyces brasiliensis	WP_013629447.1	C
	WP_013630325.1	C
	WP_013629512.1	C
	WP_013630545.1	C
	WP_013626488.1	B3
Planctomyces limnophilus	WP_013111387.1	C
	WP_013109815.1	C
	WP_013110985.1	C
	WP_013111199.1	B3
Planctomyces maris	WP_002647322.1	C
	WP_002647812.1	C
	WP_002649364.1	C
	WP_002647636.1	C
	WP_002646057.1	C
	WP_002647025.1	B2
	WP_002648234.1	B3
Rhodopirellula baltica	WP_011118454.1	C

	WP_011121964.1	C
	WP_011123966.1	C
	WP_011118661.1	C
	WP_011123282.1	B2
	WP_011121474.1	B2
Rhodopirellula maiorica	WP_008689798.1	C
	WP_008702293.1	C
	WP_008700483.1	C
	WP_008705699.1	C
	WP_008702144.1	C
	WP_008699567.1	C
	WP_008707696.1	C
	WP_008689852.1	B2
	WP_008689849.1	B2
Rhodopirellula rubra	WP_009096753.1	C
	WP_009093330.1	C
	WP_009104091.1	C
	WP_009104526.1	B2
Rhodopirellula sallentina	WP_008674585.1	B2
	WP_008676981.1	B2
Schlesneria paludicola	WP_010586473.1	C
	WP_010584564.1	C
	WP_010581694.1	B2
	WP_010585046.1	B3
Singulisphaera acidiphila	WP_015247136.1	C
	WP_015247924.1	C
	WP_015246285.1	C
	WP_015247434.1	C
	WP_015246644.1	C
	WP_015247588.1	C
	WP_015250595.1	C
	WP_015247585.1	C
	WP_015248964.1	C
	WP_015247704.1	C
	WP_015244111.1	C
	WP_015243700.1	C
	WP_020466179.1	C
	WP_015248038.1	C
	WP_015249465.	C

	WP_015245606.1	C
	WP_015244109.1	A
	WP_015249363.1	B3
	WP_015250648.1	B3
Zavarzinella formosa	WP_020475192.1	C
	WP_020470817.1	C
	WP_020471343.1	C
	WP_020471569.1	C
	WP_020474830.1	C
	WP_020473903.1	C
	WP_020471977.1	A
	WP_020475401.1	B3

Supplementary Table 5: Beta-lactamase related proteins found within planctomycetal genomes based on blastp comparison with functional proteins.

3. Supplementary Notes

Supplementary Note 1. Phylogenetic analyses

To investigate the planctomycetal cell wall composition, we first employed phylogenetic analysis to identify suitable model organisms for subsequent bioinformatic, biochemical and microscopical characterization. Since the subphylum of anammox planctomycetes differs significantly in cell biology and metabolism from all other Planctomycetes, this group was excluded from our analysis based on previously described criteria[1]. Representative strains for the phylogenetic analysis were selected based on three criteria: i) type strains according to the List of Prokaryotic Names with Standing in Nomenclature ii) sequenced strains and iii) proposed type strains. When the case of several closely related strains occurred, priority was given in order of the listed criteria to represent as much diversity as possible. The phylogenetic tree in Supplementary Fig. 1 contains these selected planctomycetal species including the yet unpublished deep branching, obligate anaerobic and halophilic strain L21-RPul-D3. The long branches of this tree lead to the suggestion of high evolutionary divergence of cultivated planctomycetal species. Based on phylogenetic analysis and unusual cell biological traits such as the lack of PG, Planctomycetes were hypothesized to be an exception to the bacterial definition (for review see[2]). While our bioinformatic analysis included all sequenced planctomycetal species shown in Supplementary Figure 1 (red), biochemical experiments focused on representative organisms of each subdivision (Supplementary Fig. 1, boxed in grey). Compared to the great phylogenetic depth of Planctomycetes, only few species are available in pure culture[1]. From those, even fewer genome sequences are available (Supplementary Fig. 1, red). Thus, in total sixteen genomes were available for bioinformatics analysis.

Supplementary Note 2. Genome mining

Comparative genomics and genome mining have been successfully employed in the past to distil a list of genes putatively involved in the unusual FtsZ-independent cell division of Planctomycetes[1]. In a further study, planctomycetal genome evolution was analysed. Using the metabolic pathway reconstruction of the KEGG database[3] some, but not all PG biosynthesis related genes were identified[4]. However, on closer inspection, this database was found to contain an almost complete set (11 of 13) of essential proteins for peptidoglycan biosynthesis in the model organism *P. limnophilus*[5]. Based on this observation we propose that the distinct phylogenetic position of the PVC superphylum and that of the Planctomycetes in particular, prevents the identification of homologous proteins by standard sequence comparison. Thus, we manually searched for PG biosynthesis related genes among sequenced Planctomycetes (Supplementary Table 2). On the other hand, a protein from *P. limnophilus* (YP_003630728.1) could be readily annotated as FlgI, the P-ring protein that anchors the bacterial flagellum in the PG layer of typical Gram-negative cells. This protein shares a significant sequence identity with FlgI from *E. coli* (WP_021570018.1: e-value 3E-28; 31% identity). Thus, planctomycetal proteins differ in amino acid sequence conservation relative to counterparts from well-studied model organisms and a careful manual inspection is required for the identification of target proteins.

In a straightforward genomic approach, one might determine whether Planctomycetes can synthesize PG from a genomic perspective by comparing all essential proteins for the synthesis of PG against the proteins encoded by the genome of sequenced Planctomycetes. However, this approach is associated with several difficulties: i) which proteins are essential for PG synthesis? ii) which query sequences of essential PG synthesis proteins should be used in comparison with Planctomycetes genes? iii) which method of comparison is appropriate? iv) how to deal with permanent draft genomes? These questions are addressed in the following.

i) Selection of essential proteins for peptidoglycan synthesis

The biosynthesis of peptidoglycan is a complex process involving approximately 20 enzymes[6]. Despite recent advances, the biosynthesis pathway is not completely understood yet (for review see[6, 7, 8]). Thus we focused on proteins that were previously experimentally shown to be essential for PG synthesis[6] and extended our query list according to the PG pathway of the KEGG database[3]. Our analysis included the murein (PG) synthesis proteins

MurA-G, the two integral membrane proteins MraY (transferase) and MviN/MurJ (flippase). In addition, we searched for penicillin binding proteins (pbp), which are targeted by beta-lactam antibiotics. Apart from their role in PG formation, these proteins are also interesting as Planctomycetes are resistant against beta-lactam antibiotics. Previous studies suggested this resistance could be contributed to the lack of PG. Instead, it could be possible that the presence of beta-lactamase enzymes is responsible for the destruction of beta-lactam antibiotics[9]. The entire set of proteins selected for the comparison with the database (Supplementary Data 2-4) is referred to as 'target proteins' in the following.

ii) Influence of query sequence selection on the identification of target proteins

In a first step, we screened for target proteins in the NCBI database employing query sequences from *E. coli*, as most of the knowledge about PG results from analysing this bacterium[8]. We found that members of the genus *Planctomyces* encode proteins for the complete PG biosynthesis pathway, while most other Planctomycetes either lacked all or several target proteins (Supplementary Data 1). Positive results could be verified by reciprocal BLAST analysis. In the past others and we stopped at this point[1, 10], assuming that the screening outcome concurs with the dogma that Planctomycetes lack PG[9]. However, the phylogenetic distance between Enterobacteriaceae and Planctomycetes is huge. In addition, long branches in the planctomycetal phylogenetic tree (Supplementary Fig. 1), which are only supported by few genomes, suggest that Planctomycetes are undersampled in sequence databases. Taken together, this could hinder protein identification based on pairwise sequence alignments. Thus, for further screening attempts, we used PG synthesis related protein sequences identified in the model organism *P. limnophilus*[11] as queries. With this strategy, we found more, but still not all target proteins in the sixteen analysed genomes (Supplementary Data 2).

iii) Position-specific iterative BLAST search for target proteins

Since pairwise sequence alignments seemed to be insufficient in the quest for planctomycetal PG biosynthesis homologues we employed the PSI-BLAST algorithm at five iterations[12]. Using this approach, we identified most of the missing target proteins in all sequenced Planctomycetes (Supplementary Data 3). Yet, PSI-BLAST failed to detect certain target proteins that were determined with our previous strategy and verified through reverse BLAST

analysis (see *i)* above). For example, the MurF protein from *P. brasiliensis* (YP_004270973.1) was identified employing the conventional blastp algorithm (e-value: 3E-88; 38% identity; Supplementary Data 2). This might be caused by structurally conserved protein domains of MurC-F[6]. In general, except for *Planctomyces*, *Schlesneria* and *Phycisphaera*, the proteins MurC-F were identified with a rather weak e-value and a similarity of 13%- 31% and thus considered to be MurC-F-like proteins. However, as such proteins might be functional redundant to some degree this most likely does not affect the PG synthesis capability.

iv) Incomplete planctomycetal genomes

The genomes of *B. marina*, *G. obscuriglobus*, *P. maris*, *R. maiorcia*, *R. sallentina*, *R. rubra*, *S. paludicola* and *Z. formosa* are permanent draft genomes and complete chromosome sequences are not available. Therefore, it cannot be excluded that some gene sequences were not identified because the respective draft genome assemblies did not cover the corresponding genome regions. For example, in our analysis we could not detect MurB in *R. sallentina* and MraY in *Z. formosa.*

Supplementary Note 3. Genome mining for beta-lactamase enzymes

The genome sequence of *Planctomyces limnophilus* was found to contain 13 putative beta-lactamase encoding genes[15]. This prompted us to employ a rigid bioinformatic screen for beta-lactamase encoding genes in the selected planctomycetal genomes (Supplementary Table 1). Through comparison with functional proteins belonging to the molecular classes A-D and subclasses B1-B3 (selected based on ref.[16], Supplementary Table 3), we found planctomycetal genomes to encode between 1-19 putative beta-lactamase related proteins (Supplementary Tables 4+5). Thus at least one putative beta-lactamase gene is present in each of the analysed genomes.

Although we predicted seven and six beta-lactamase encoding genes in the genomes of *Planctomyces maris* and *Pirellula marina* (=*Blastopirellula marina*), respectively, these organisms have previously been demonstrated to lack beta-lactamase activity[17]. However, the employed nitrocefin assay is not reliable, as it produces false negative results and fails to detect the activity in all classes of beta-lactamases[18]. Our own repetition of these experiments showed a weak beta-lactamase activity in L21-RPul-D3 cells whereas none was detected in *P. limnophilus* (Supplementary Fig. 3). We predicted L21-RPul-D3 to encode one, and *P. limnophilus* to encode at least four putative beta-lactamases (Supplementary Table 4). Thus, as others concluded before, the nitrocefin assay can prove beta-lactamase activity, but a negative result does not exclude the presence of beta-lactamase enzymes. The resistance against beta-lactam antibiotics caused by a specific enzyme activity might be further supported by a 'gene dosage' effect: strain L21-RPul-D3 (one putative beta-lactamase gene, Supplementary Table 4) can withstand carbenicillin concentrations up to 100 mg/l. In contrast, *P. limnophilus* (four putative beta-lactamase genes, Supplementary Table 4) can grow with a tenfold higher concentration (1 g/l). Thus, the resistance of L21-RPul-D3 can be broken at drug concentrations, which are not yet toxic to all other Planctomycetes. In conclusion, resistance to beta-lactam antibiotics is more likely to be caused by enzymes that can be saturated rather than by a lack of PG.

4. Supplementary Discussion

The biosynthesis of PG involves approximately 20 enzymatic reactions and is far from being entirely understood (ref.[6, 7, 8]). Hence, all assumptions about a minimal list of genes required for PG production are problematic. In addition, sequences encoding the four Mur ligases (MurC-F) are known to be highly diverse among various bacterial species (15% -22% identity). On the other hand, the four proteins share three structural highly conserved domains that function in the nonribosomal peptide bond formation in PG synthesis[13]. Thus, correct annotation of MurC-F with sequence based bioinformatics methods is difficult. In addition, fusion of genes involved in PG synthesis such as *murE*/*murF* or *murC*/*ddl* for example, could prevent identification[14]. This might also be the case for Planctomycetes, where identification for the respective Mur ligases failed the threshold (see above). Likewise, the fact that eight of the 16 analysed planctomycetal genomes are only represented as permanent draft genomes might hinder the identification not only of the PG synthesis, but of genes in general. With the exception of the fully sequenced genome of *R. baltica* where no *pbp* and no typical flippase protein (MviN/ MurJ) could be identified, only Planctomycetes with permanent draft genomes lacked proteins for PG synthesis (see Supplementary Table 2 for summary). This explains why previous attempts could have fallen short to identify all genes required for PG synthesis in Planctomycetes[4]. However, despite such limitations, our extended approach employing blastp combined with PSI-BLAST at 5 iterations provided evidence that, from a genomic perspective, PG production in most if not all Planctomycetes is possible.

In all analysed Planctomycetes, except *G. obscuriglobus*, *I. pallida* and *Z. formosa,* MurE, the diaminopimelate ligase, was identified with an e-value < 6,00E-13 and a sequence coverage >24% (Supplementary Table 2). This indicates that Planctomycetes, as most Gram-negative bacteria, might employ diaminopimelic acid for crosslinking stem peptides in their PG sacculus. However, we were not able to identify any *pbp* for *R. rubra*, *R. sallentina* and *R. baltica* but succeeded for *R. maiorica*. Moreover, it appears that *R. baltica* lacks a typical flippase protein that transfers PG precursors from the cytoplasm to the periplasm.

In summary, using different query sequences and blastp combined with the PSI-BLAST algorithm, we identified nearly all essential PG biosynthesis related genes in the sixteen investigated Planctomycetes (Supplementary Table 2). Our findings were confirmed employing reciprocal blastp analysis of identified putative planctomycetal PG synthesis proteins against the *E. coli* proteome (Supplementary Data 4). Thus, the bioinformatic analyses provide evidence that most, if not all Planctomycetes are able to synthesize peptidoglycan. In particular, most Planctomycetes encode penicillin binding proteins, which

are targets for beta-lactam antibiotics. Accordingly, the resistance of all so far analysed Planctomycetes against beta-lactam antibiotics cannot be explained with the absence of PG as indicated in the literature[9].

5. Supplementary References

1. Jogler C*, et al.* Identification of proteins likely to be involved in morphogenesis, cell division, and signal transduction in Planctomycetes by comparative genomics. *Journal of bacteriology* **194**, 6419-6430 (2012).

2. Fuerst JA, Sagulenko E. Beyond the bacterium: *Planctomycetes* challenge our concepts of microbial structure and function. *Nature reviews* **9**, 403-413 (2011).

3. Kanehisa M, Goto S, Sato Y, Kawashima M, Furumichi M, Tanabe M. Data, information, knowledge and principle: back to metabolism in KEGG. *Nucleic acids research* **42**, D199-205 (2014).

4. Guo M*, et al.* Genomic evolution of 11 type strains within family Planctomycetaceae. *PloS one* **9**, e86752 (2014).

5. Jogler M, Jogler C. Towards the development of genetic tools for Planctomycetes. In: *New Models for Cell Structure, Origins and Biology: Planctomycetes.* (eds Fuerst J). Springer (2013).

6. Lovering AL, Safadi SS, Strynadka NC. Structural perspective of peptidoglycan biosynthesis and assembly. *Annu Rev Biochem* **81**, 451-478 (2012).

7. Bugg TD, Braddick D, Dowson CG, Roper DI. Bacterial cell wall assembly: still an attractive antibacterial target. *Trends Biotechnol* **29**, 167-173 (2011).

8. Desmarais SM, De Pedro MA, Cava F, Huang KC. Peptidoglycan at its peaks: how chromatographic analyses can reveal bacterial cell wall structure and assembly. *Molecular microbiology* **89**, 1-13 (2013).

9. König E, Schlesner H, Hirsch P. Cell-wall studies on budding bacteria of the *Planctomyces/ Pasteuria* group and on a *Prosthecomicrobium* sp. *Archives of microbiology* **138**, 200-205 (1984).

10. Pilhofer M*, et al.* Characterization and evolution of cell division and cell wall synthesis genes in the bacterial phyla Verrucomicrobia, Lentisphaerae, Chlamydiae, and Planctomycetes and phylogenetic comparison with rRNA genes. *Journal of bacteriology* **190**, 3192-3202 (2008).

11. Jogler C, Glöckner FO, Kolter R. Characterization of *Planctomyces limnophilus* and development of genetic tools for its manipulation establish it as a model species

for the phylum *Planctomycetes*. *Applied and environmental microbiology* **77**, 5826-5829 (2011).

12. Altschul SF*, et al.* Gapped BLAST and PSI-BLAST: a new generation of protein database search programs. *Nucleic acids research* **25**, 3389-3402 (1997).

13. Bouhss A, Mengin-Lecreulx D, Blanot D, van Heijenoort J, Parquet C. Invariant amino acids in the Mur peptide synthetases of bacterial peptidoglycan synthesis and their modification by site-directed mutagenesis in the UDP-MurNAc:L-alanine ligase from *Escherichia coli*. *Biochemistry* **36**, 11556-11563 (1997).

14. McCoy AJ, Maurelli AT. Characterization of Chlamydia MurC-Ddl, a fusion protein exhibiting D-alanyl-D-alanine ligase activity involved in peptidoglycan synthesis and D-cycloserine sensitivity. *Molecular microbiology* **57**, 41-52 (2005).

15. Labutti K*, et al.* Complete genome sequence of *Planctomyces limnophilus* type strain (Mu 290). *Stand Genomic Sci* **3**, 47-56 (2010).

16. Bush K. The ABCD's of beta-lactamase nomenclature. *Journal of infection and chemotherapy : official journal of the Japan Society of Chemotherapy* **19**, 549-559 (2013).

17. Claus H, Martin HH, Jantos CA, Konig H. A search for beta-lactamase in chlamydiae, mycoplasmas, planctomycetes, and cyanelles: Bacteria and bacterial descendants at different phylogenetic positions and stages of cell wall development. *Microbiological Research* **155**, 1-6 (2000).

18. Pitkälä A, Salmikivi L, Bredbacka P, Myllyniemi AL, Koskinen MT. Comparison of tests for detection of beta-lactamase-producing staphylococci. *Journal of clinical microbiology* **45**, 2031-2033 (2007).
